# Glycemic control predicts the Brain-derived neurotrophic factor levels in diabetic neuropathy patients with a diabetic duration of at least 5 years: A cross-sectional study

**DOI:** 10.12688/f1000research.172834.1

**Published:** 2025-11-26

**Authors:** Maria Suryani, Maria Agustina Ermi Tri Sulistiyowati, Medina Sianturi

**Affiliations:** 1 Nursing, Sekolah Tinggi Ilmu Kesehatan Elisabeth Semarang, Semarang, Central Java, Indonesia

**Keywords:** BDNF; HbA1c; Diabetic duration; Diabetes; Diabetic neuropathy

## Abstract

**Background:**

Diabetic neuropathy is one of the complications of diabetes that occurs due to poor glycemic control. Brain-derived neurotrophic factor (BDNF) levels can increase when patients are first diagnosed with type 2 diabetes mellitus and can change in response to glycemic control conditions throughout the course of the disease. However, the correlation between glycemic control and BDNF remains unclear. The objective of this study was to investigate whether glycemic control can predict the BDNF levels in patients with diabetic neuropathy, based on diabetic duration.

**Methods:**

A cross-sectional study was conducted on 80 patients with diabetic neuropathy who were treated at a clinic in Central Java. We use glycated hemoglobin (HbA1c) levels as a parameter of glycemic control, which were measured according to the National Glycohemoglobin Standardization Program. BDNF serum levels were evaluated using the Enzyme-Linked Immunosorbent Assay (ELISA) method in the laboratory. Analysis was performed using the Spearman rank correlation and linear regression tests.

**Results:**

The results of the study show that HbA1c levels in diabetic neuropathy patients with DM <5 years and ≥5 years differed significantly (9.50% vs. 8.9%, p=0.049), while BDNF levels did not differ significantly (1136.98 pg/mL vs 948.07 pg/mL, p=0.172). There is a positive correlation between HbA1c and BDNF levels in patients with a diabetic duration of at least 5 years (r=0,423, p=0,007). There is no correlation between HbA1c and BDNF levels in patients with a diabetic duration of less than 5 years. The HbA1c level was a significant predictor of BDNF serum level in patients with DM duration of at least 5 years (B=120.317, 95% CI for B: 6.103 – 234.532, p=0.039).

**Conclusions:**

The HbA1c levels significantly predict BDNF levels in patients with diabetic neuropathy who have had a diabetic duration of at least 5 years.

## Introduction

Approximately 50% of T2DM patients are found to have diabetic neuropathy complications. ^1^ An additional year of diabetic duration and a 1% increase in glycated hemoglobin (HbA1c) levels increase a person’s risk of developing diabetic neuropathy by one-fold. ^2^ Patients with diabetic neuropathy experience damage to large or small nerve fibers, or even both, which may cause a range of symptoms, including pain, numbness, muscle weakness, and autonomic dysfunction. ^3^ Patients with more severe diabetic neuropathy will experience a variety of signs and symptoms due to the extensive damage to these large and small nerves. ^4^

Prolonged hyperglycemia can cause inflammation and oxidative stress, resulting in damage to peripheral nerve fibers in patients with diabetic neuropathy. ^5, 6^ The process of nerve damage involves the accumulation of oxidative and pro-inflammatory substances in the nerves. ^6^ The presence of inflammation and oxidative stress in T2DM patients affects the production of brain-derived neurotrophic factor (BDNF) levels, which ultimately changes BDNF levels. ^7^ BDNF levels may change throughout the course of T2DM, with an increase in BDNF may occur in prolonged hyperglycemia. ^8^ BDNF is a neurotrophin that plays an important role in the central nervous system and systemic or peripheral inflammatory conditions in T2DM with neuropathy. ^9, 10^ BDNF functions in preventing nerve cell damage, maintaining nerve cell survival, and playing a role in nerve cell plasticity. ^11^ A decrease in BDNF levels can further worsen neuropathy. ^12^

According to a previous study, patients with diabetes have higher BDNF levels compared to healthy people. ^11^ Additionally, another study also found a positive correlation between insulin resistance and fasting blood sugar levels, with BDNF levels in patients with diabetes mellitus. ^7^ BDNF levels continued to increase with increasing pain severity in patients with diabetic neuropathy. ^9, 13^ However, the correlation between glycemic control and BDNF levels is still unclear. The other study has shown that the HbA1c level is not correlated with BDNF levels. ^14^ In addition, the other previous study also found that BDNF levels decrease in patients with diabetes mellitus and diabetic neuropathy. ^15^ The duration of diabetes likely influences the relationship between glycemic control and serum BDNF levels. ^16^ This study aims to investigate whether glycemic control can predict BDNF levels in patients with diabetic neuropathy, based on diabetic duration. The diabetic duration will be divided into <5 years and ≥5 years.

## Methods

### Study design

A cross-sectional study was conducted among patients with diabetic neuropathy in a primary health care facility in Central Java, Indonesia.

### Study population

The study was conducted on 80 participants with diabetic neuropathy, who were treated at a primary health care facility in Central Java, Indonesia. The inclusion criteria were a minimum of 1 month of suffering from T2DM since diagnosis by a doctor, and having diabetic neuropathy. The exclusion criteria were having a stroke, an active ulcer, fracture in the foot.

### Data collection

Data collection was done between July 2025 and August 2025. The duration of diabetes was measured by asking patients about the time between their diagnosis and the time of data collection in years. The diabetic duration was categorized into <5 years and ≥5 years. We use HbA1c as a parameter of glycemic control, while the BDNF serum level is used to measure the BDNF level. Venous blood samples were collected to measure BDNF and HbA1c levels. The blood samples were taken at the same time as the measurement of the duration of diabetes. HbA1c levels were determined by analyzing blood serum in a laboratory using an HPLC technique with the GNSP, following current institutional biosafety protocols, while BDNF serum levels were determined using Human BDNF enzyme-linked immunosorbent assay kit (CAT#RE2848H-96 wells 31.25-20000 pg/mL, Reedbiotech Ltd, Wuhan, China) according to the manufacturer’s instructions. We executed BDNF procedures at the GAKI laboratory of Diponegoro University, and HbA1c procedures at the PRODIA laboratory. We have also collected the patient characteristics such as age, body mass index (BMI), gender, working status, and diabetic neuropathy severity. The diabetic neuropathy symptom score (NSS) and diabetic neuropathy examination (DNE) were used as diabetic severity parameters. DNE score was defined as the accumulation score of the result measurement consists of eight items, including two tests of muscle strength, one of the reflex Achilles, and five tests of sensation. The min-max score is 0-16. The score was determined by doing a physical examination. The NSS score was defined as the accumulation score of the result measurement, consisting of sixteen items about the symptoms of neuropathy. The higher the score indicated the more severe the neuropathy. All collected data were evaluated for missing data during data entry. No missing data was found in this study.

### Data analysis

The data were analyzed using SPSS 23.00. Categorical data were presented in the form of frequencies and percentages, while numerical data were presented in means and standard deviations. Kolmogorov-Smirnov was used to examine the distribution of numerical data. The Spearman Correlation test was used to examine the correlation between glycemic control and BDNF levels based on diabetic duration. Variables with p-values less than 0.25 in the Spearman analysis were then entered into a multivariate linear regression model. The linear regression analysis, by model, was used to examine the predictors of BDNF levels. Statistical significance was established using a threshold of p = 0.05.

### Ethical approval

The study procedure was reviewed and approved by the external ethics committee of the University Widya Husada Semarang (31/EC/LPPM/UWHS/VII/2025). The study complied with the Helsinki Declaration. Written informed consent was obtained from participants of the study prior to the study.

## Results

Table 1 shows that there are significant differences in age, employment status, and HbA1c values between patients who have had diabetes for less than 5 years and those who have had it for at least 5 years. The patients with a diabetic duration of at least 5 years were older and had lower HbA1c levels than patients with a diabetic duration of less than 5 years. The BDNF level of patients with a diabetic duration of at least 5 years was lower than that of patients with a diabetic duration of less than 5 years; however, the difference in BDNF level was not significant.

**Table 1.** Characteristics of the participants.

Characteristics	Diabetic duration <5 years	Diabetic duration ≥5 years	P
Age (year): mean (SD)	57.6 ± 9.6	62.63 ± 10.66	0.026 ^a^
BMI (Kg/m ^2^): mean ± SD	25.97 ± 5.49	27.48 ± 8.01	0.400 ^a^
Gender, number (%)			
Male	10 (62.5)	6 (37.5)	0.264 ^b^
Female	30 (46.9)	34 (53.1)	
Working status, number (%)			
Employee	19 (65.5)	10 (34.5)	0.036 ^b^
Unemployee	21 (41.2)	30 (58.8)	
Medication (%)			
Metformin or glimepiride	27 (69.2)	12 (30.8)	0.007 ^d^
Metformin and glimepiride	7 (26.9)	19 (73.1)	
Insulin and metformin/glimepiride	1 (20)	4 (80)	
Diabetic neuropathy duration (year), mean ± SD	1.90 ± 2.95	3.51 ± 4.25	0.024 ^a^
Diabetic neuropathy severity			
DNE score	10.18 ± 1.67	9.53 ± 2.58	0.313 ^a^
NSS score	7.97 ± 2.52	7.83 ± 2.71	0.689 ^a^
BDNF serum levels (pg/ml), mean ± SD	1136.98 ± 813.59	948.07 ± 69.98	0.172 ^a^
HbA1c levels (%), mean ± SD	9.55 ± 2.75	8.49 ± 1.87	0.049 ^c^

^a^ Mann-Whitney U test,

^b^ Chi-square test,

^c^ Paired T test,

^d^ Kolmogorov-Smirnov test.

Table 2 shows that there is a significant positive correlation between glycemic control and BDNF levels in patients who have had diabetes for at least 5 years (r = 0.423, p = 0.007). There is no significant correlation between glycemic control and BDNF levels in patients who have had diabetes for less than 5 years (r = 0.217, p = 0.178).

**Table 2.** Correlation between glycemic control and BDNF levels in patients with different durations of T2DM.

	Diabetic duration <5 years		Diabetic duration ≥5 years	
	p	r	p	r
Glycemic control	0.178	0.217	0.007	0.423
Age	0.488	0.113	0.269	- 0.167
BMI	0.016	-0.37	0.591	-0.088
Gender	0.294	-0.170	0.195	-0.209
Working status	0.175	0.219	0.339	-0.155
Medication	0.095	0.267	0.748	0.052
Diabetic neuropathy duration	0.489	0.113	0.935	0.013
DNE score	0.401	0.136	0.018	0.372
NSS score	0.583	0.091	0.487	0.113

Table 3 shows that HbA1c level was not a predictor of BDNF in diabetic neuropathy patients with a duration of diabetes of less than 5 years, whereas in the group of patients with a duration of diabetes of at least 5 years, glycemic control was a significant predictor by 8.3% (B = 120.317, 95% CI for B: 6.103 – 234.532, p = 0.039).

**Table 3.** Predictors of BDNF levels in multiple regression analysis, by model.

Diabetic duration	Model	Variable	B Coefficient	p-value	95% CI
<5 years	1 Adj. R ^2^ = 20.8%	Glycemic control	45.210	.314	-44.593 – 135.013
		BMI	-32.867	.151	-78.266 – 12.532
		Working status	453.731	.057	-15.228 – 922.690
		Medication	230.489	.050	-.268 – 461.247
	Final Adj. R ^2^ = 20.7%	BMI	-39.566	.073	-82.955 – 3.823
		Working status	480.745	.043	15.040 – 946.449
		Medication	231.568	.049	0.907 – 462.229
≥5 years	1 Adj. R ^2^ = 9.8%	Gender	105.483	0.730	-508.562 – 719.527
		Glycemic control	97.163	0.103	-20.457 – 214.782
		DNE score	-70.387	0.116	-159.018 – 18.244
	2 Adj. R ^2^ = 11.9%	Glycemic control	96.590	0.100	-19.469 – 212.649
		DNE score	-66.377	0.120	-150.778 – 18.024
	Final Adj. R ^2^ = 8.3%	Glycemic control	120.317	0.039	6.103 – 234.532

## Discussion

This study investigated whether glycemic control can predict the BDNF levels in patients with diabetic neuropathy, based on diabetic duration. Patient characteristics showed that the majority of patients with diabetic neuropathy were elderly, female, and obese (>25 kg/m ^2^). The diabetic neuropathy can occur in T2DM patients aged less than 40, suffering from T2DM for less than 1 year, in all genders, whether obese or not. ^17^ However, diabetic neuropathy will increase in males, older adults, and longer diabetic duration. ^17^

The characteristics of patients with diabetic neuropathy with a duration of less than 5 years and at least 5 years did not differ significantly in terms of gender, BMI, diabetes medication, severity of diabetic neuropathy, duration of diabetic neuropathy, or BDNF levels. Although not significantly different, these various characteristics can influence BDNF levels in patients. The previous study has shown that women, obesity, diabetes medications such as metformin and glimepiride, and higher severity of diabetic neuropathy can alter BDNF levels. ^18^

The results showed that the characteristics of diabetic neuropathy patients with diabetes duration of less than 5 years and at least 5 years differed significantly in terms of age, occupation, and HbA1c levels. Diabetic neuropathy patients with a diabetes duration of at least 5 years had a higher mean age, were more unemployed, and had higher mean HbA1c levels compared to diabetic neuropathy patients with a diabetes duration of less than 5 years. The previous study has shown that age, activity, and HbA1c levels can influence a person’s BDNF levels. ^18– 20^

This study did not show significant differences in BDNF serum levels in diabetic neuropathy patients with varying durations of diabetes. However, BDNF serum levels in diabetic neuropathy patients with longer durations of diabetes were lower than in patients with shorter durations. Bivariate test results showed that only HbA1c levels and DNE scores correlated with serum BDNF levels in patients with diabetes duration of at least 5 years. Multivariate analysis showed that HbA1c levels could predict BDNF serum levels. Previous studies have evaluated the correlation between HbA1c and BDNF, but these studies were conducted in diabetic patients without neuropathy. ^7, 15^ Consistent with previous studies that showed higher HbA1c levels indicate higher BDNF levels. ^7^ This correlation was found in newly diagnosed diabetic patients and in patients who had diabetes for an average of 4 years. ^7^

In this study, patients with diabetic neuropathy who had suffered for at least 5 years had uncontrolled glycemic levels of 8.49%. When the HbA1c levels are high for a long time in patients, the complications of diabetic neuropathy can occur. ^2^ In this study, the patients had experienced diabetic neuropathy for 3.5 years, meaning that for the previous five years, they had experienced high HbA1c levels that triggered damage to their peripheral nerves. Inflammation and oxidative stress occur when blood sugar levels are uncontrolled, resulting in the accumulation of pro-inflammatory and oxidative substances in the peripheral nerves. ^5, 6^ This triggers an increase in BDNF levels to improve glucose metabolism, overcome inflammation, and reduce oxidative stress. Furthermore, BDNF production functions to overcome peripheral nerve damage because BDNF plays a role in nerve cell plasticity and maintains nerve cell survival. ^3, 6, 9^

Conversely, no correlation was found between HbA1c and BDNF levels in patients with a duration of diabetes of less than 5 years. Although no correlation was found in this group, this study showed that HbA1c and BDNF levels were elevated. Elevated HbA1c and BDNF levels were also found in patients with diabetic neuropathy with a longer duration. The study shows that the worse a patient’s glycemic control, the higher their BDNF levels, and vice versa. This occurs because with higher hyperglycemia, more BDNF is produced to improve glucose metabolism. However, this study appears to indicate that the duration of hyperglycemia exposure in patients can influence the relationship between HbA1c and BDNF levels.

In addition, the results of the DNE examination revealed peripheral nerve damage with a mean score of 9.53 out of a maximum of 16 in patients with diabetes for at least 5 years. The DNE examination revealed damage to large and small nerve fibers, which function in muscle movement, tendon reflexes, vibration sensation, pain sensation, touch sensation, and position sense. ^21^ Bivariate analysis results in patients with diabetes for at least 5 years showed a positive correlation between the severity of diabetic neuropathy and BDNF levels. This suggests that increased BDNF synthesis in response to nerve damage prevents further nerve damage that could worsen the patient’s neuropathy. The previous study has shown that patients with diabetic neuropathy who exhibit signs of hypersensitivity to pain have increased BDNF levels. ^16^ Increased pain levels in patients with neuropathy are a potential indicator of neuropathy severity. ^16^ This increase in pain generally occurs at night. This study found that patients also complained of leg pain, which worsened at night.

The study also found that the bivariate test results indicate that BMI is correlated with BDNF levels, but the multivariate test results indicate that diabetes treatment and occupation are related to BDNF levels in patients with diabetic neuropathy with a duration of diabetes of less than 5 years. Obesity can worsen the patient’s insulin resistance. ^22, 23^ Previous studies have shown that insulin resistance is associated with BDNF levels. ^7^ It appears that the presence of insulin resistance that occurs early due to obesity can explain the relationship between BMI and BDNF levels in patients. ^7, 11^ Patients with T2DM will receive antidiabetic medicine to lower their blood sugar levels. ^24^ The patient’s HbA1c level indicates that the patient’s glycemic control is in the poor category and requires treatment. ^24^ The majority of patients receive antidiabetic medication, namely metformin and glimepiride. The previous study has shown that metformin and glimepiride increase BDNF levels, in addition to lowering blood sugar levels. ^25^ Furthermore, the study found that the majority of patients with diabetic neuropathy with a duration of less than 5 years were still active. Activity can affect BDNF levels. It appears that activity can stimulate BDNF production.

This study has a limitation. There was a potential bias in determining diabetes duration because patients were newly diagnosed with T2DM, often after they experienced complications. The study was carried out with a small number of participants; thus, a study with a larger number of participants is needed in future studies. This study also only used HbA1c as a parameter of glycemic control.

The study implies that HbA1c can be used as a predictor of BDNF levels, especially in patients who have diabetic neuropathy with a diabetic duration of at least 5 years. The health providers should monitor the HbA1c level regularly in patients with diabetic neuropathy. The future study can evaluate the other glycemic control parameters.

### Reporting guidelines

This study was reported in accordance with the STROBE guidelines.

## Data Availability

Zenodo: Dataset corresponding to the scientific paper “Glycemic control predicts the Brain-derived neurotrophic factor levels in diabetic neuropathy patients with a diabetic duration of at least 5 years: A cross-sectional study” https://doi.org/10.5281/zenodo.17540079 ^26^ The project contains the following underlying data: Data set diabetic neuropathy study.xlsx-raw data of participants’ characteristics, BDNF, and HbA1c. Data are available under the terms of the Creative Commons Zero “No right reserved” data waiver (CCO v1.0 Universal)
