# Glycemic control predicts the Brain-derived neurotrophic factor levels in diabetic neuropathy patients with a diabetic duration of at least 5 years: A cross-sectional study

**DOI:** 10.12688/f1000research.172834.4

**Published:** 2026-06-09

**Authors:** Maria Suryani, Maria Agustina Ermi Tri Sulistiyowati, Medina Sianturi

**Affiliations:** 1 Nursing, Sekolah Tinggi Ilmu Kesehatan Elisabeth Semarang, Semarang, Central Java, Indonesia

**Keywords:** BDNF; HbA1c; Diabetic duration; Diabetes; Diabetic neuropathy

## Abstract

**Background:**

Diabetic neuropathy is one of the complications of diabetes that occurs due to poor glycemic control. Brain-derived neurotrophic factor (BDNF) levels can increase when patients are first diagnosed with type 2 diabetes mellitus and can change in response to glycemic control conditions throughout the course of the disease. However, the correlation between glycemic control and BDNF remains unclear. The objective of this study was to investigate whether glycemic control can predict the BDNF levels in patients with diabetic neuropathy, based on diabetic duration.

**Methods:**

A cross-sectional study was conducted on 8 patients with diabetic neuropathy who were treated at a clinic in Central Java. We use glycated hemoglobin (HbA1c) levels as a parameter of glycemic control, which were measured according to the National Glycohemoglobin Standardization Program. BDNF serum levels were evaluated using the Enzyme-Linked Immunosorbent Assay (ELISA) method in the laboratory. Analysis was performed using ANCOVA tests.

**Results:**

Together, HbA1c levels, diabetic duration, and interactions between diabetic duration and HbA1c explained 9.9% of variability in BDNF levels (p=0.046). However, HbA1c levels explained 8.1% of the variability in BDNF levels (p = 0.011), with only minor contributions from diabetic duration and interaction them.

**Conclusions:**

The HbA1c levels significantly explained the variability in BDNF levels in patients with diabetic neuropathy regardless of diabetic duration.

## Introduction

Approximately 50% of T2DM patients are found to have diabetic neuropathy complications. ^1^ An additional year of diabetic duration and a 1% increase in glycated hemoglobin (HbA1c) levels increase a person’s risk of developing diabetic neuropathy by one-fold. ^2^ Patients with diabetic neuropathy experience damage to large or small nerve fibers, or even both, which may cause a range of symptoms, including pain, numbness, muscle weakness, and autonomic dysfunction. ^3^ Patients with more severe diabetic neuropathy will experience a variety of signs and symptoms due to the extensive damage to these large and small nerves. ^4^

Prolonged hyperglycemia can cause inflammation and oxidative stress, resulting in damage to peripheral nerve fibers in patients with diabetic neuropathy. ^5, 6^ The process of nerve damage involves the accumulation of oxidative and pro-inflammatory substances in the nerves. ^6^ The presence of inflammation and oxidative stress in T2DM patients affects the production of brain-derived neurotrophic factor (BDNF) levels, which ultimately changes BDNF levels. ^7^ BDNF levels may change throughout the course of T2DM, with an increase in BDNF may occur in prolonged hyperglycemia. ^8^ BDNF is a neurotrophin that plays an important role in the central nervous system and systemic or peripheral inflammatory conditions in T2DM with neuropathy. ^9, 10^ BDNF functions in preventing nerve cell damage, maintaining nerve cell survival, and playing a role in nerve cell plasticity. ^11^ A decrease in BDNF levels can further worsen neuropathy. ^12^

According to a previous study, patients with diabetes have higher BDNF levels compared to healthy people. ^11^ Additionally, another study also found a positive correlation between insulin resistance and fasting blood sugar levels, with BDNF levels in patients with diabetes mellitus. ^7^ BDNF levels continued to increase with increasing pain severity in patients with diabetic neuropathy. ^9, 13^ However, the correlation between glycemic control and BDNF levels is still unclear. The other study has shown that the HbA1c level is not correlated with BDNF levels. ^14^ In addition, the other previous study also found that BDNF levels decrease in patients with diabetes mellitus and diabetic neuropathy. ^15^ The duration of diabetes likely influences the relationship between glycemic control and serum BDNF levels. ^16^ This study aims to investigate whether glycemic control can predict BDNF levels in patients with diabetic neuropathy, based on diabetic duration. The diabetic duration will be divided into <5 years and ≥5 years.

## Methods

### Study design

A cross-sectional study was conducted among patients with diabetic neuropathy in a primary health care facility in Central Java, Indonesia.

### Study population

**Figure 1.**
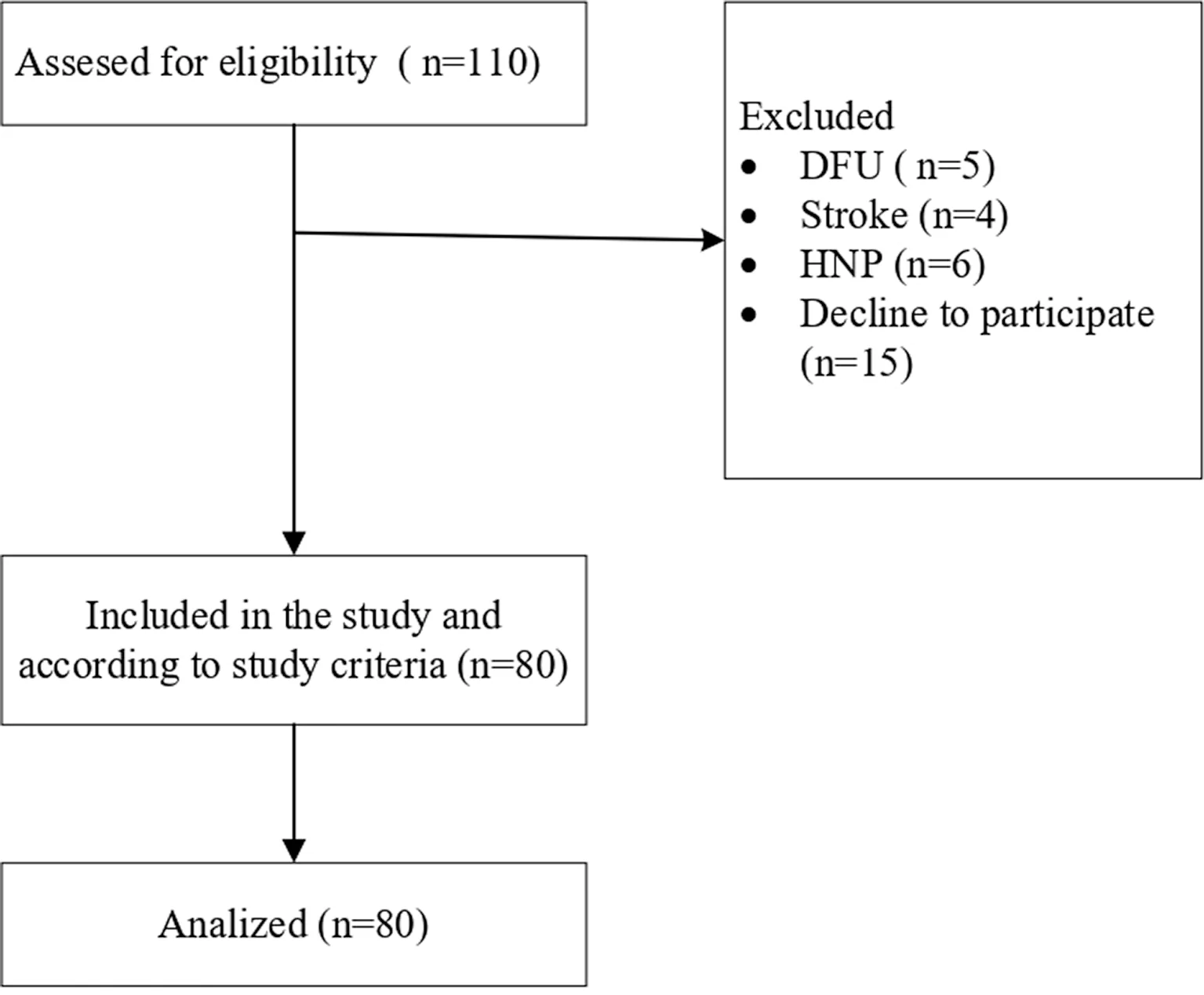
STROBE flow diagram.

The study was conducted on 80 participants with diabetic neuropathy, who were treated at a primary health care facility in Central Java, Indonesia. The inclusion criteria were a minimum of 1 month of suffering from T2DM since diagnosis by a doctor, and having diabetic neuropathy. The exclusion criteria were having a stroke, an active ulcer, fracture in the foot. Figure 1 shows that outlines the overarching process to be followed in the conduct of this study; from identifying relevant participant at title and abstract stage to data collected and analysis.

### Data collection

Data collection was done between July 2025 and August 2025. The duration of diabetes was measured by asking patients about the time between their diagnosis and the time of data collection in years. The diabetic duration was categorized into <5 years and ≥5 years. We use HbA1c as a parameter of glycemic control, while the BDNF serum level is used to measure the BDNF level. Venous blood samples were collected to measure BDNF and HbA1c levels. The blood samples were taken at the same time as the measurement of the duration of diabetes. HbA1c levels were determined by analyzing blood serum in a laboratory using an HPLC technique with the GNSP, following current institutional biosafety protocols, while BDNF serum levels were determined using Human BDNF enzyme-linked immunosorbent assay kit (CAT#RE2848H-96 wells 31.25-20000 pg/mL, Reedbiotech Ltd, Wuhan, China) according to the manufacturer’s instructions. We executed BDNF procedures at the GAKI laboratory of Diponegoro University, and HbA1c procedures at the PRODIA laboratory. We have also collected the patient characteristics such as age, body mass index (BMI), gender, working status, and diabetic neuropathy severity. The diabetic neuropathy symptom score (NSS) and diabetic neuropathy examination (DNE) were used as diabetic severity parameters. DNE score was defined as the accumulation score of the result measurement consists of eight items, including two tests of muscle strength, one of the reflex Achilles, and five tests of sensation. The min-max score is 0-16. The score was determined by doing a physical examination. The NSS score was defined as the accumulation score of the result measurement, consisting of sixteen items about the symptoms of neuropathy. The higher the score indicated the more severe the neuropathy. All collected data were evaluated for missing data during data entry. No missing data was found in this study.

### Data analysis

The data were analyzed using SPSS 23.00. Mann-Whitney U test, Chi square, and independent t-test were used to compare the characteristic of participant between patient who have diabetic duration <5 years and ≥5 years. Spearman rank test was used to examine the correlation between glycemic control and BDNF level. The regression analysis ANCOVA, by model was used to examine the predictors of BDNF levels (Glycemic control, diabetic duration and interaction between glycemic control and diabetic duration). Statistical significance was established using a threshold of p < 0.05.

### Ethical approval

The study procedure was reviewed and approved by the external ethics committee of the University Widya Husada Semarang (31/EC/LPPM/UWHS/VII/2025). The study complied with the Helsinki Declaration. Written informed consent was obtained from participants of the study prior to the study.

## Results

Table 1 shows that there are significant differences in age, working status, medication, and HbA1c levels between patients who have had diabetes for less than 5 years and those who have had it for at least 5 years. The BDNF level of patients with a diabetic duration of at least 5 years was lower than that of patients with a diabetic duration of less than 5 years; however, the difference in BDNF level was not significant.

**Table 1.** Characteristics of the participants.

Characteristics	Diabetic duration <5 years	Diabetic duration ≥5 years	P
Age (year): mean (SD)	57.6 ± 9.6	62.63 ± 10.66	0.026 ^a^
BMI (Kg/m ^2^): mean ± SD	25.97 ± 5.49	27.48 ± 8.01	0.400 ^a^
Gender, number (%)			
Male	10 (62.5)	6 (37.5)	0.264 ^b^
Female	30 (46.9)	34 (53.1)	
Working status, number (%)			
Employee	19 (65.5)	10 (34.5)	0.036 ^b^
Unemployee	21 (41.2)	30 (58.8)	
Medication (%)			
Metformin or glimepiride	27 (69.2)	12 (30.8)	0.007 ^b^
Metformin and glimepiride	7 (26.9)	19 (73.1)	
Insulin and metformin/glimepiride	1 (20)	4 (80)	
Diabetic neuropathy duration (year), mean ± SD	1.90 ± 2.95	3.51 ± 4.25	0.024 ^a^
Diabetic neuropathy severity			
DNE score	10.18 ± 1.67	9.53 ± 2.58	0.313 ^a^
NSS score	7.97 ± 2.52	7.83 ± 2.71	0.689 ^a^
BDNF serum levels (pg/ml), mean ± SD	1136.98 ± 813.59	948.07 ± 69.98	0.172 ^a^
HbA1c levels (%), mean ± SD	9.55 ± 2.75	8.49 ± 1.87	0.049 ^c^

^a^ Mann-Whitney U test.

^b^ Chi-square test.

^c^ Independent T test.

Table 2 shows that there is a significant correlation between glycemic control and BDNF (r = 0.312, p = 0.005).

**Table 2.** Correlation between HbA1c and BDNF level (n = 80).

	BDNF	
	r	p
**HbA1c**	0.312	0.005

p: Spearman test.

Table 3 shows that, together, HbA1c level, diabetic duration, and the interactions between them explained ~10% of variability in BDNF levels (overall η ^2^ = 0.099; p = 0.046) when analyzed by GLM regression. However, HbA1c levels provided most of the regression model’s explanatory power (partial η ^2^ = 0.081; p = 0.011), with only minor contributions coming from diabetic duration (partial η ^2^ = 0.003; p = 0.617) and the duration *HbA1c interaction (partial η ^2^ = 0.004, p = 0.571).

**Table 3.** Predictors of BDNF levels in multiple regression analysis, by model.

	Sum of Square	df	Mean square	F	p	η ^2^
Corrected Model	4479133.84	3	1493044.614	2.790	0.046	0.099
HbA1c	3670941.455	1	3670941.455	6.860	0.011	0.081
Diabetic duration	135232.373	1	135232.373	0.253	0.617	0.003
Diabetic duration * HbA1c	173435.384	1	173435.384	0.324	0.571	0.004
Error	40670978.107	76	535144.449			
Corrected Total	45150111.950	79				

R Square = 0.099, R Adj = 0.064.

Analyses were conducted by ANCOVA and were adjusted for other variables in the table.

## Discussion

The study investigated whether glycemic control, as measured by HbA1c parameters, can predict BDNF levels in diabetic neuropathy patients based on the diabetic duration. Diabetic neuropathy will increase in patients with longer diabetic duration. ^17^ The study found that together, HbA1c levels, diabetic duration, and interactions between diabetic duration and HbA1c explained 9.9% of variability in BDNF levels. However, only HbA1c levels explained the variability in BDNF levels at 8.1%, with only minor contributions from diabetic duration and interaction them. It means that HbA1c level can explain BDNF level regardless of diabetic duration. In line with previous studies that found that HbA1c levels can predict BDNF levels. ^18– 20^

It seems that when hyperglycemia occurs in diabetic neuropathy patients, there will be damage to nerve cells due to the accumulation of oxidative and pro-inflammatory substances. Inflammation and oxidative stress occur when blood sugar levels are uncontrolled, resulting in the accumulation of pro-inflammatory and oxidative substances in the peripheral nerves, both large and small nerve fibers. ^5, 6^ Increasing BDNF synthesis in response to nerve damage prevents further nerve damage that could worsen the patient’s neuropathy. BDNF functions in preventing nerve cell damage, maintaining nerve cell survival, and playing a role in nerve cell plasticity. ^3, 6, 9^ The DNE examination revealed the condition of large and small nerve fibers, which function in muscle movement, tendon reflexes, vibration sensation, pain sensation, touch sensation, and position sense. ^21^ This study found that the results of DNE examination of patients showed the presence of nerve cell damage in all patients with various lengths of diabetic duration. This whole process demonstrates how HbA1c levels can explain the variability in BDNF levels in diabetic neuropathy patients.

The study divided the diabetic durations into less than 5 years and at least 5 years. There are significant differences in characteristics such as age, working status, medication, and HbA1c levels in patients with different diabetic durations. Patients with diabetes who were less than 5 years old had a lower mean age, were more unemployed, and were taking a single anti-diabetic medicine in the form of metformin or glimepiride compared to diabetic neuropathy patients with a diabetic duration of at least 5 years. It seems that differences in these ages, activity, and anti-diabetic medicine make up for differences in HbA1c levels. ^22– 25^ In this study, patients with diabetic neuropathy who had suffered for at least 5 years had uncontrolled glycemic levels of 8.49%, while those who had suffered for less than 5 years had glycemic control of 9.55%. This suggests that diabetic duration is related to HbA1c levels or that there is an interaction of diabetic duration with HbA1c.

The finding of a patient’s HbA1c levels associated with diabetic duration indirectly allows for the prediction of diabetic duration, and the interaction between diabetic duration and HbA1c can also predict BDNF levels. This study found that, together with diabetic duration, HbA1c levels, and the interaction between diabetic duration and HbA1c levels can significantly explain the variation of BDNF levels, even though only 9.9%. However, of all these predictors, only HbA1c can significantly explain the variability in BDNF levels. The study also showed that there are still other predictors not described in this study that may be able to explain the variability in BDNF levels.

This study has a limitation. There was a potential bias in determining diabetic duration because patients were newly diagnosed with T2DM, often after they experienced complications. Thus, we divided diabetic duration into two categories: less than 5 years and at least 5 years. The study was carried out with a small number of participants; thus, a study with a larger number of participants is needed in future studies.

The study implies that HbA1c can explain the variability in BDNF level, even though only 8.1%, especially in patients who have diabetic neuropathy regardless of diabetic duration. The health providers should monitor the HbA1c level regularly in patients with diabetic neuropathy. The future study can evaluate the other predictors of BDNF level with a large number of participants.

## Data Availability

Zenodo: Dataset corresponding to the scientific paper “Glycemic control predicts the Brain-derived neurotrophic factor levels in diabetic neuropathy patients with a diabetic duration of at least 5 years: A cross-sectional study” https://doi.org/10.5281/zenodo.17540079. ^26^ The project contains the following underlying data: Data set diabetic neuropathy study.xlsx-raw data of participants’ characteristics, BDNF, and HbA1c. Data are available under the terms of the Creative Commons Zero “No right reserved” data waiver (CCO v1.0 Universal) This study was reported in accordance with the STROBE guidelines.
