# Supplementary material for: Glycemic control predicts the Brain-derived neurotrophic factor levels in diabetic neuropathy patients with a diabetic duration of at least 5 years: A cross-sectional study
Source: F1000Res. 2026 Jun 9;14:1312. Originally published 2025 Nov 26. [Version 4] doi: 10.12688/f1000research.172834.4 (PMC13596705; doi:10.12688/f1000research.172834.4)
Supplement: Peer review history

# Reviewer report: Reviewer response for version 4

**DOI:** 10.5256/f1000research.203067.r492257

**Published:** 2026-06-12

**Authors:** Eric Siegel

**Affiliations:** 1 University of Arkansas for Medical Sciences, Little Rock, AR, USA

The Results section of the Abstract contains one small, but important spelling error. The phrase "interaction them" should be corrected to read "interaction term".

Is the work clearly and accurately presented and does it cite the current literature?

Partly

If applicable, is the statistical analysis and its interpretation appropriate?

No

Are all the source data underlying the results available to ensure full reproducibility?

Yes

Is the study design appropriate and is the work technically sound?

Partly

Are the conclusions drawn adequately supported by the results?

No

Are sufficient details of methods and analysis provided to allow replication by others?

Partly

Reviewer Expertise:

Applied Biostatistics

I confirm that I have read this submission and believe that I have an appropriate level of expertise to confirm that it is of an acceptable scientific standard.

# Reviewer report: Reviewer response for version 3

**DOI:** 10.5256/f1000research.200446.r488296

**Published:** 2026-05-28

**Authors:** Eric Siegel

**Affiliations:** 1 University of Arkansas for Medical Sciences, Little Rock, AR, USA

Please make the following change to the Results part of the Abstract:

 Old paragraph: 

 Together, HbA1c levels, diabetic duration, and interactions between diabetic duration and HbA1c explained 9.9% of variability in BDNF levels and 6.4% after adjusted (p=0.046). However, only HbA1c levels explained 8.3% of the variability in BDNF levels (p = 0.011).

 Please change to:

 New paragraph:

 Together, HbA1c levels, diabetic duration, and the interaction between them explained 9.9% of variability in BDNF levels (p=0.046). However, HbA1c levels  explained 8.3% of the variability in BDNF levels (p = 0.011), with only minor contributions from diabetic duration and the interaction term.  

 In Table 3, please make the following two changes.

 (1) Change the eta-square for HbA1c from 0.083 to 0.081. 

 (2) Delete the value of the Mean Square for Corrected Total.

 In the Results part that discusses Table 3, please change the paragraph as follows:

 Old paragraph:

 Table 3 shows that together HbA1c level, diabetic duration, and interactions between diabetic duration and HbA1c explained 9.9% of variability in BDNF levels and 6.4% after adjusted (p = 0.046). However, only HbA1c levels explained 8.3% of the variability in BDNF levels.

 New paragraph:

 Table 3 shows that, together, HbA1c level, diabetic duration, and the interaction between them explained ~10% of variability in BDNF levels (overall η ^2^ = 0.099; p = 0.046) when analyzed by GLM regression. However, HbA1c levels provided most of the regression model’s explanatory power (partial η ^2^ = 0.081; p = 0.011), with only minor contributions coming from Diabetic duration (partial η ^2^ = 0.003; p = 0.617) and the duration*HbA1c interaction (partial η ^2^ = 0.004; p = 0.571).

Is the work clearly and accurately presented and does it cite the current literature?

Partly

If applicable, is the statistical analysis and its interpretation appropriate?

No

Are all the source data underlying the results available to ensure full reproducibility?

Yes

Is the study design appropriate and is the work technically sound?

Partly

Are the conclusions drawn adequately supported by the results?

No

Are sufficient details of methods and analysis provided to allow replication by others?

Partly

Reviewer Expertise:

Applied Biostatistics

I confirm that I have read this submission and believe that I have an appropriate level of expertise to confirm that it is of an acceptable scientific standard, however I have significant reservations, as outlined above.

# Reviewer report: Reviewer response for version 2

**DOI:** 10.5256/f1000research.197388.r476298

**Published:** 2026-04-21

**Authors:** Eric Siegel

**Affiliations:** 1 University of Arkansas for Medical Sciences, Little Rock, AR, USA

The article is much improved. 

 Results from the ANCOVA regression analysis are provided in Table 3. 

 However, Table 3 is not complete. It is missing things. 

 Table 3 does contain the Mean Squares, F-statistics, p-values, and eta-squares. Those are good. Please keep them.

 Missing from Table 3 are the Sums of Squares and the Degrees of Freedom (DF) for each row in the table. Please add those things to Table 3. The SPSS output should have each row's Sum of Squares and DF. Each row's Sum of Squares provides the numerator in that row's calculation of eta-square.

 Also missing from Table 3 is the "Total" row that contains the Total Sum of Squares and the Total DF. Please add that row of Totals to Table 3. The SPSS output should have the Total Sum of Squares and Total DF. The Total Sum of Squares provides the denominator in the calculations of the eta-squares.

 Furthermore, the authors misinterpret the meaning of the eta-squares. The eta-squares do not measure accuracy. They measure how much of the data's variation (the Total Sum of Squares) is "explained" by the factor in the model. In that sense, they are exactly like R-squares and Partial R-squares from regression analysis. 

 For example, the eta-square of 0.099 for corrected model does *not* mean 9.9% accuracy at predicting BDNF with HbA1c +DM Duration +Interaction together. It means that, together, HbA1c +DM Duration +Interaction "explain" 9.9% of the variability in BDNF. "Explain" is not the same as predict. 

 Similarly, the eta-square of 0.083 for HbA1c does *not* mean that HbA1c can predict BDNF at 8.3%. It means that the HbA1c component of the corrected model "explains" 8.3% of the variability in BDNF. And that's when the corrected model has the other components as well as HbA1c. And again, "explain" is not the same as predict. 

 In Summary, (1) Table 3 is missing important pieces of information, and (2) the eta-squares of Table 3 are wrongly interpreted as predictive instead of explanatory.

Is the work clearly and accurately presented and does it cite the current literature?

Partly

If applicable, is the statistical analysis and its interpretation appropriate?

No

Are all the source data underlying the results available to ensure full reproducibility?

Yes

Is the study design appropriate and is the work technically sound?

Partly

Are the conclusions drawn adequately supported by the results?

No

Are sufficient details of methods and analysis provided to allow replication by others?

Partly

Reviewer Expertise:

Applied Biostatistics

I confirm that I have read this submission and believe that I have an appropriate level of expertise to state that I do not consider it to be of an acceptable scientific standard, for reasons outlined above.

# Response

**Published:** 2026-04-27

**Authors:** Maria Suryani

**Affiliations:** Nursing, Sekolah Tinggi Ilmu Kesehatan Elizabeth Semarang, Semarang, Central Java, Indonesia

Dear Eric Siegel

 Thank you for your suggestion. The revised version of the manuscript incorporates the corrections suggested by the reviewer. Changes have been made to abstract section especially in result and conclusion. Changes have been made also to research result in table 3, and discussion according to new interpretation of result of the study in table 3.  Thank you

 Best regards

 Maria Suryani (author)

# Reviewer report: Reviewer response for version 1

**DOI:** 10.5256/f1000research.190594.r439043

**Published:** 2025-12-30

**Authors:** Eric Siegel

**Affiliations:** 1 University of Arkansas for Medical Sciences, Little Rock, AR, USA

The authors assert that the correlation between glycemic control and BDNF levels is still unclear. They also hypothesize that the duration of diabetes likely influences the relationship between glycemic control and serum BDNF levels, but they did not speculate how. Therefore, the authors seek to investigate whether glycemic control can predict BDNF levels in patients with diabetic neuropathy, based on diabetic duration. The Diabetes Mellitus durations (DMDs) are dichotomized as less than 5 years versus 5 years or longer, and patients are divided into two groups according to that dichotomization.

 The study is a cross-sectional study of eligible patients seen in a single health-care facility during a one-month time window. The shortness of the time window is a positive thing. HbA1c levels and BDNF levels were obtained from blood samples drawn during the time window. When they had their blood drawn, patients were asked how many years ago they were diagnosed with their diabetes. I suspect that the accuracy of the patients’ answers was good when the diabetes diagnosis was recent, but poor when the diabetes diagnosis was more than a few years back. This probably explains why the authors chose to divide patients into 2 groups based on a 5-year cutpoint for DMDs. I probably would have done the same.  

 My concern is with how the authors did their analysis. It appears they did subgroup analyses of HbA1c versus BDNF level subgrouped by DMD group, using spearman correlation within each subgroup. This is the wrong approach. A much better approach would have been to perform regression analysis on all 80 patients using a regression model that has BDNF level as the dependent or “outcome” variable, and has DMD Group and HbA1c Level as the two independent or “predictor” variables. This regression model is an Analysis of Covariance (ANCOVA) model with DMD Group as the categorical predictor and HbA1c Level as the continuous predictor.

 One assumption of the ANCOVA model is that the relationship between BDNF and HbA1c is equal in both DMD groups. The authors will need to test the validity of that assumption. To do so, the authors will need to expand the ANCOVA model by adding to it the “DMD-x-HbA1c Interaction” as a third predictor variable. And then they will need to test the statistical significance of the DMD-x-HbA1c Interaction. If the interaction is statistically significant at p<0.05, then that result will provide strong support for the authors’ hypothesis that “the duration of diabetes likely influences the relationship between glycemic control and serum BDNF levels”. If the interaction is non-significant, say, with P>0.10, then that result will tend to falsify the authors’ hypothesis, and falsification of hypotheses are a good thing. If the interaction is “almost significant”, i.e., 0.05≤P≤0.10, then that is the worst-case scenario, but the authors still could spin it as weakly supportive of the authors’ hypothesis, but requiring additional studies with larger sample sizes to confirm.

 Table 2: I do not see the value of doing univariable spearman correlations of BDNF with Table 2’s factors subgrouped by DMD. I think it would have more value to remove the subgrouping by DMD and re-do the spearman correlations on all 80 patients considered as one group.

 Furthermore, I see strong risk of having a falsely significant result somewhere in Table 2. If a test’s significance level is P<0.05, then the test’s false-positive rate is alpha=0.05 by definition. With 18 tests in Table 2, each at alpha=0.05 per test, the expected number of false-positive results in Table 2 is 18*0.05=0.90 or almost 1. Which result in Table 2 is the false-positive result? There is no way to tell. The two best ways to avoid this quandary are (1) reduce the number of tests in Table 2, and/or (2) reduce each test’s significance level down to P<0.01.

 In Table 3, there is no value in building multivariable regression models subgrouped by DMD group. It would be much, much better to build multivariable regression models on all 80 patients in the study, and to include DMD Group as a predictor variable in those multivariable models. If the authors are worried that DMD group affects the size of the B coefficient for BDNF versus Glycemic control, then the statistically valid way to test for that possibility is to include the DMD-x-HbA1c interaction term in the multivariable models so that one can test its significance.

 Finally, the conclusions are framed in terms of predicting BDNF levels in patients with diabetic neuropathy. If the goal is to predict whether glycemic control in an individual patient can predict that patient’s BDNF levels, then spearman correlations, t-tests, and multivariable regressions are wholly inadequate to the task. To investigate patient-level prediction ability, one would need to explore ROC curves and more advanced methods.

 Other Concerns.

 The paper needs a STROBE flow diagram that shows (1) how many patients were initially evaluated, (2a) how many were excluded for having a stroke, (2b) how many were excluded for having an active ulcer, (2c) how many were excluded for a fracture in the foot, and (3) how many were included in the final analysis. If the STROBE flow diagram were also to show how many patients fell into the DMD <5-year group vs how many patients fell into the DMD ≥5-year group, that would be a plus.

 Table one indicates that the two DMD groups had exactly 40 patients per group, i.e., the two DMD groups were equal in size. If DMD was simply dichotomized at <5 vs ≥5 years, then we would expect the two DMD groups to be unequal in size because sample size would be a random variable. Did the authors do anything to control how many patients fell into each DMD group? Such as, impose a maximum on the number in one group?

 The authors say that Kolmogorov-Smirnov was used to examine the distribution of numerical data. Examine it for what? For goodness of adherence to the Normal (or other parametric) Distribution? Or for whether the two DMD groups had different distributions? The Kolmogorov-Smirnov test can be used to examine both. Please add some detail.

 The authors say that Kolmogorov-Smirnov was used to examine the distribution of numerical data. But in Table 1, the footnotes say it was used to compare Medication usages between DMD groups. Medication usage is categorical, not numeric.

 In Table 1, the footnotes say that the Paired T test was used to compare HbA1c levels between DMD groups. The Paired T test is the wrong test for this.  

 In Table 2, the authors put the column of P-values first and the column of correlation coefficients second. This is backwards. This puts significance first and science second. The p-value by itself has no intrinsic meaning. All of its meaning comes from the correlation coefficient to which it is attached. The correlation coefficient is the thing that has intrinsic meaning. It is where the science lies. The size of the correlation coefficient is what we evaluate when we interpret the result scientifically. So please, next time, put science ahead of significance. Put the correlations first and the p-values second.

 Regarding Table 3, I see several different types of “Adj. R ^2^” results including two types with the word “Final” in them. That implies that the authors did some variable selection *after* they entered variables “with p-values less than 0.25 in the Spearman analysis” into the models, but the authors do not say what else they did after they entered those variables. More detail is needed.

Is the work clearly and accurately presented and does it cite the current literature?

Partly

If applicable, is the statistical analysis and its interpretation appropriate?

No

Are all the source data underlying the results available to ensure full reproducibility?

Yes

Is the study design appropriate and is the work technically sound?

Partly

Are the conclusions drawn adequately supported by the results?

No

Are sufficient details of methods and analysis provided to allow replication by others?

Partly

Reviewer Expertise:

Applied Biostatistics

I confirm that I have read this submission and believe that I have an appropriate level of expertise to state that I do not consider it to be of an acceptable scientific standard, for reasons outlined above.

# Response

**Published:** 2026-03-11

**Authors:** Maria Suryani

**Affiliations:** Nursing, Sekolah Tinggi Ilmu Kesehatan Elizabeth Semarang, Semarang, Central Java, Indonesia

The revised version of the manuscript incorporates the corrections suggested by the reviewers. Changes have been made to abstract section especially in method and result. Changes have been made also to data analysis, research result tables, and discussion according to new result of the study. We have added STROBE diagram.

# Reviewer report: Reviewer response for version 1

**DOI:** 10.5256/f1000research.190594.r439051

**Published:** 2025-12-27

**Authors:** Ummu Muntamah

**Affiliations:** 1 Universitas Ngudi Waluyo, Ungaran, Central Java, Indonesia

- The manuscript is generally well written and clearly presented. However, the use of the term “predicts” may overstate the findings given the cross-sectional design. In addition, although relevant literature is cited, the manuscript would benefit from incorporating more recent studies and systematic reviews to strengthen the state of the art, particularly regarding the dual role of BDNF in diabetic neuropathy.
- Several studies have shown decreased BDNF in chronic diabetes. These contradictions have not been critically addressed (e.g., differences in disease stage, age, or comorbidities).
- The discussion would have been stronger if the authors had: distinguished the initial compensation phase versus long-term BDNF depletion, associated a duration of ≥5 years with the possibility of neurotrophic exhaustion.
- Need to be strengthened with literature from the last 5 years. Most of the references appear to be non-recent literature (many studies predate 2020). There are minimal references to: recent systematic reviews or meta-analyses, large studies related to BDNF as a biomarker for diabetic neuropathy, literature from the last 5 years on neurotrophins and diabetes complications.
- There is no discussion of recent literature on: the role of BDNF as a compensatory marker versus a marker of nerve damage, longitudinal dynamics of BDNF in chronic diabetes.  This makes the state of the art section feel insufficiently robust.
- The design is appropriate for association analysis, but not ideal for predictive or mechanistic claims. Inclusion and exclusion criteria were clearly explained. All participants had diabetic neuropathy, so the population was relatively homogeneous. However: The sampling method (consecutive, convenience, random) was not explained.There was no sample size calculation, so statistical power cannot be determined.
- The study design is appropriate for exploring associations between glycemic control and BDNF levels in diabetic neuropathy. The laboratory methods and statistical analyses are generally sound. However, the cross-sectional nature of the study limits causal or predictive interpretations. The manuscript would benefit from clearer justification of the diabetes duration cut-off, reporting of regression assumptions, and consideration of biological confounders affecting BDNF levels.
- In general, the methods and analyses are reported clearly enough to understand what the researchers did, but not sufficiently to allow for exact replication by other researchers. Conceptual replication is still possible, but some important technical details are missing or not explicitly reported.
- The methods and statistical analyses are described clearly at a general level; however, several methodological details required for full replication are missing. Additional information on sampling procedures, blood collection protocols, ELISA assay handling, and regression diagnostics would strengthen the reproducibility of the study.
- The statistical analyses are generally appropriate and adequately performed. However, the interpretation occasionally overstates the findings, particularly through the use of predictive language in a cross-sectional design. Reporting of regression diagnostics and a more cautious interpretation of effect size would strengthen the statistical rigor of the manuscript.
- The manuscript does not provide access to the underlying individual-level data supporting the reported results. While summary statistics are presented, the absence of raw data, a data availability statement, and statistical code limits full reproducibility. The authors are encouraged to deposit anonymized source data in a public repository or clarify conditions for data access.
- the conclusions are generally supported by the results presented. However, some statements overstate the findings, particularly regarding predictive and mechanistic implications. Reframing the conclusions to emphasize associations rather than causality would improve alignment with the study design and results.

Is the work clearly and accurately presented and does it cite the current literature?

Partly

If applicable, is the statistical analysis and its interpretation appropriate?

Yes

Are all the source data underlying the results available to ensure full reproducibility?

Partly

Is the study design appropriate and is the work technically sound?

Yes

Are the conclusions drawn adequately supported by the results?

Partly

Are sufficient details of methods and analysis provided to allow replication by others?

Partly

Reviewer Expertise:

non-communicable disease, innovation of health promotion

I confirm that I have read this submission and believe that I have an appropriate level of expertise to confirm that it is of an acceptable scientific standard.

# Response

**Published:** 2026-03-11

**Authors:** Maria Suryani

**Affiliations:** Nursing, Sekolah Tinggi Ilmu Kesehatan Elizabeth Semarang, Semarang, Central Java, Indonesia

Thank you for your suggestion to our article. The revised version of the manuscript incorporates the corrections suggested by the reviewer. We still use predictor term according to new statistical analysis. We have used the updated references according to our topic.
